# Effect of Low-Level Laser Stimulation on EEG

**DOI:** 10.1155/2012/951272

**Published:** 2012-09-02

**Authors:** Jih-Huah Wu, Wen-Dien Chang, Chang-Wei Hsieh, Joe-Air Jiang, Wei Fang, Yi-Chia Shan, Yang-Chyuan Chang

**Affiliations:** ^1^Department of Biomedical Engineering, Ming Chuan University, No. 5 Deming Road, Gweishan Township, Taoyuan 333, Taiwan; ^2^Department of Sports Medicine, China Medical University, No. 91 Hsueh-Shih Road, Taichung City 404, Taiwan; ^3^Department of Photonic and Communication Engineering, Asia University, Taichung 413, Taiwan; ^4^Department of Bio-Industrial Mechatronics Engineering, National Taiwan University, No. 1, Section 4, Roosevelt Road, Taipei 106, Taiwan; ^5^Department of Neurology, Min-Sheng General Hospital, No. 168 Jin-Kuo Road, Taoyuan City, Taoyuan County 330, Taiwan

## Abstract

Conventional laser stimulation at the acupoint can induce significant brain activation, and the activation is theoretically conveyed by the sensory afferents. Whether the insensible low-level Laser stimulation outside the acupoint could also evoke electroencephalographic (EEG) changes is not known. We designed a low-level laser array stimulator (6 pcs laser diode, wavelength 830 nm, output power 7 mW, and operation frequency 10 Hz) to deliver insensible laser stimulations to the palm. EEG activities before, during, and after the laser stimulation were collected. The amplitude powers of each EEG frequency band were analyzed. We found that the low-level laser stimulation was able to increase the power of alpha rhythms and theta waves, mainly in the posterior head regions. These effects lasted at least 15 minutes after cessation of the laser stimulation. The amplitude power of beta activities in the anterior head regions decreased after laser stimulation. We thought these EEG changes comparable to those in meditation.

## 1. Introduction

Low-frequency stimulation, including electricity, sound, magnetic field, light, and laser, is widely used in different areas of medicine [1]. The EEG activity can be affected by different stimulation modalities, not only electrostimulation [2], sound, and light (visual) [3], but also magnetic stimulation [4]. Red stroboscopic light can cause rapid and powerful build-up of alpha rhythms in the occipital cortex [5]. Stimulation with variable frequency photostimulation created from red light-emitting diodes (LEDs) caused the alpha rhythms of subjects being highly influenced by different stimulation frequency [6]. As for laser, there were only a few reports on EEG changes to laser stimulation. Litscher et al. found that the cerebral blood flow velocity and the amplitudes of 40 Hz cerebral oscillations of the brain can be enhanced by stimulating either the eyes with red light or the acupoints with laser as well as needle acupuncture [7]. However, EEG changes to somatic stimulation with low-level laser (LLL) have not been reported.

LLL has been applied to clinical medicine for a long time. LLL biostimulation was first proposed in 1969 by a Hungarian scientist, Professor Mester, and then LLL therapy became popular allover the world after 1970 [8]. There were many clinical applications with LLL therapy, for instance to improve postoperative care in peripheral nerve surgeries [9] and to increase the wound tensile strength [10]. LLL has also been used to relieve a variety of chronic pains [11–13]. 

The combined use of EEG and functional magnetic resonance image (fMRI) has been proposed as an effective tool to study brain dynamics with both high temporal and high spatial resolution [14]. In spatial analysis of brain fMRI, the brain activity affected by stimulating the Yongquan (KI1) acupoint at the dorsum of the subjects' foot was validated in our previous study [15]. The aim of the present study was to investigate the EEG activities affected by the stimulation of near infrared laser array stimulator (LAS) operated at 10 Hz in temporal analysis (EEG). The LAS was applied to stimulate the palm, and then the variation of EEG activities before, during, and after the laser irradiation was analyzed. 

## 2. Subjects and Methods

### 2.1. Participants

Prior to the trial, the study protocol was approved by the Institutional Ethics Committee of Min-Sheng General Hospital. Each participant was required to give written informed consent. This study was directed in conformity with the guidelines in the Helsinki Declaration. 

Forty male normal healthy subjects were included. The subjects were randomly assigned to two groups: 20 testers in the laser group (irradiated by LAS) and 20 in the control group (sham LAS). Exclusion criteria included (a) having a history of psychiatric disorders, for example, major depression, substance abuse, and schizophrenic or paranoid disorder; (b) having cardiopulmonary disease; (c) receiving medication.

### 2.2. Laser

In this study, an array of laser diodes arranged in a triangle shape were used [16]. The laser diodes of LAS were mounted in a transparent acrylic holder to isolate the laser diodes from the skin of the tester. The LAS consists of 6 laser diodes (LDs) (manufactured by Advanced Chips & Products Corp., Hillside, NJ; wavelength 830 nm; maximum output power 30 mW). Each LD was set at 7 mW output for minimum stimulation in this study, and the operational frequency is adjustable from 1 to 20 Hz, duty cycle 50%. The electronic circuit and optical design in this system have been reported elsewhere [16]. The light of laser diode without any collimated lens is a stripe shape due to the different divergence angle in horizontal (10°) and vertical (30°) directions. The area of the laser light is approximately equal to 14.8 mm^2^ at 10 mm distance. Thus, the dosage would be approximately 20 Joules/cm^2^ for 10-minute treatment; it is insensible on operation. 

### 2.3. Procedure

The “single-blind randomized trial” was used in this study. The subjects were unaware which group they were in. Each subject sat in an armchair and was then required to put his left palm on the LAS device. Participants were instructed to relax, follow the open-eyes directive, and withhold from any movements. In the laser group, the laser diodes were turned on for 10 minutes and not turned on in the control group. In the beginning, the testers were relaxed for five minutes to stabilize physiological parameters. The ongoing EEG was then recorded with eyes opened in three stages (6 sessions): before stimulation (baseline 5 min, session 1), during stimulation (laser stimulation, 10 min, session 2 and session 3), after stimulation (poststimulation, 15 min, and sessions 4, 5, and 6).

### 2.4. Control

The low-power infrared laser diode is ideally suited for a single-blind study since the laser light is invisible and emits no heat or any other detectable indication. The testers were randomly divided into two operational modes: laser group who received the real laser stimulation and control group who received the sham laser. The control group had the same procedure as the laser group, but the laser stimulator was not turned on. 

### 2.5. EEG Recording and Measurement

During experiment, the left palm of the tester was put on the LAS. A 16-channel electroencephalograph (Neurofax model EEG-1000, NIHON KOHDEN) was used in this study. The band pass was set at 0.53–70.0 Hz. The variation of EEG potential was recorded on the scalp with Ag/AgCl recording electrodes. Electrode placement was arranged following international 10–20 system. Bipolar recording to take potential between Fp2-F4, F4-C4, C4-P4, P4-O2, Fp1-F3, F3-C3, C3-P3, P3-O1, Fp2-F8, F8-T4, T4-T6, Fp1-F7, F7-T3, and T3-T5 was performed. An additional ECG was recorded by placing electrodes on both hands. Six sessions of EEG data were collected. Each set of EEG data (5 min epoch) was treated with fast Fourier transform (FFT) analysis to obtain the EEG band power (*μ*v^2^) in the following 4 frequency bands: delta (0.5–3.5 Hz); theta (4–7 Hz); alpha (8–13 Hz); beta (13–50 Hz). The data obtained from bipolar recording were shown in time domain; for each session, the four band powers of valid epochs were averaged, and FFT maps of different session were performed by the software (Neurofax ver. 05-80). The mean and standard deviation of calculated values were expressed as “mean ± SD.”

### 2.6. Statistical Analysis

Two-tailed paired *t*-test was applied to compare the difference of EEG band power before and after LAS stimulation from Fp2-F4, F4-C4, C4-P4, P4-O2, Fp1-F3, F3-C3, C3-P3, P3-O1, Fp2-F8, F8-T4, T4-T6, Fp1-F7, F7-T3, and T3-T5 electrodes. All the statistical analyses were executed with the SPSS software. A statistical significance was recognized as *P* value < 0.05.

## 3. Result

**Figure 1:**
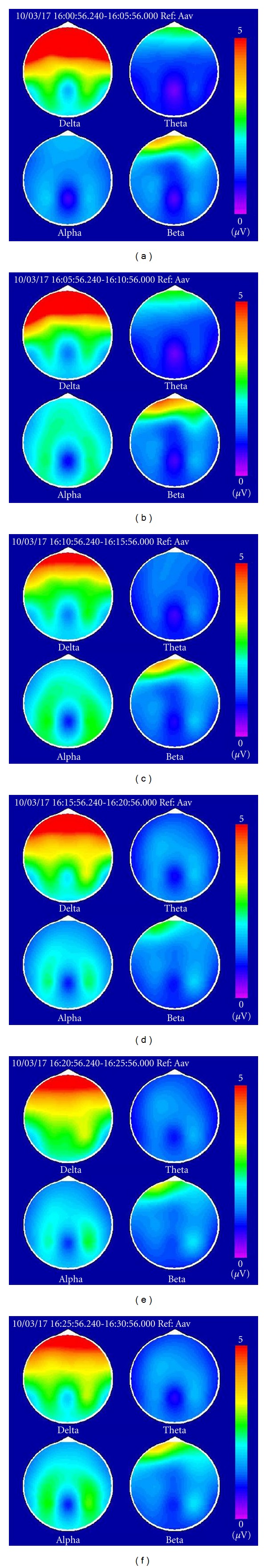
(a)–(f) The power spectrum maps of EEG in the six sessions of one subject with low-level laser stimulation.

**Figure 2:**
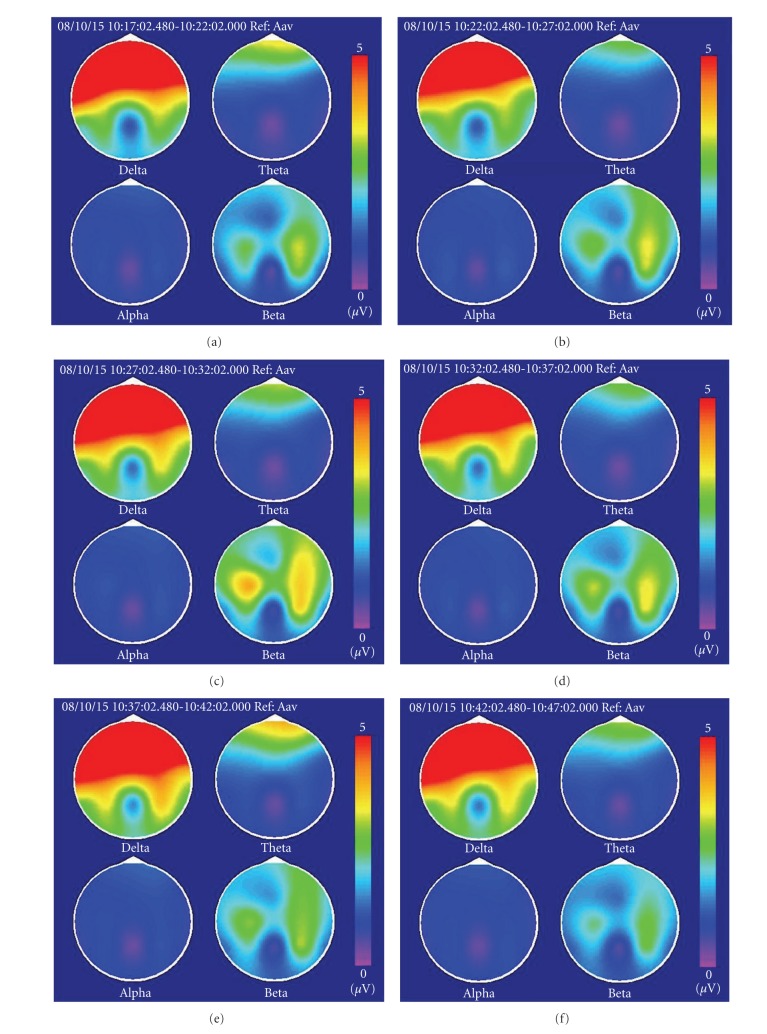
(a)–(f) The power spectrum maps of EEG in the six sessions of one subject with no low-level laser stimulation.

The mean ± SD of age in the laser and the control group was 20.20 ± 0.58 and 20.90 ± 0.70 years, respectively. There was no significant difference between these two groups (*P* > 0.05). As showed in Table 1, the average EEG power of alpha rhythm (8–13 Hz) at session 1 showed no significant differences between the two groups. The power spectrum maps of the six sessions in one subject of the laser group were shown in Figures 1(a)–1(f).  Figure 1(a) is the baseline stage; Figures 1(b) and 1(c) are stimulation stages. The average power of alpha (8–13 Hz) is apparently increased. In the poststimulation stages (sessions 4–6), activation of alpha power is clearly maintained in these sessions, as shown in Figures 1(d)–1(f). Meanwhile, the power spectrum maps of the six sessions of one subject in the control group were shown in Figures 2(a)–2(f). The average power of alpha (8–13 Hz) did not have any apparent change.

After the collection of data, we compared and analyzed the values of the laser and control groups. The comparison between these two groups in each session was done for the alpha band. Due to weak laser stimulation (almost 7 mW for each laser), the differences between these two groups are not significant meanings in all recording regions (*P* > 0.05), as shown in Table 2, comparison between the two groups in session 2. There were no significant differences between the two groups in other sessions; these data were omitted here for concise reason. On the other hand, there were no significant differences between these two groups in each session in delta, theta, and beta band (*P* > 0.05). These data were omitted here for concise reason too. 

**Figure 3:**
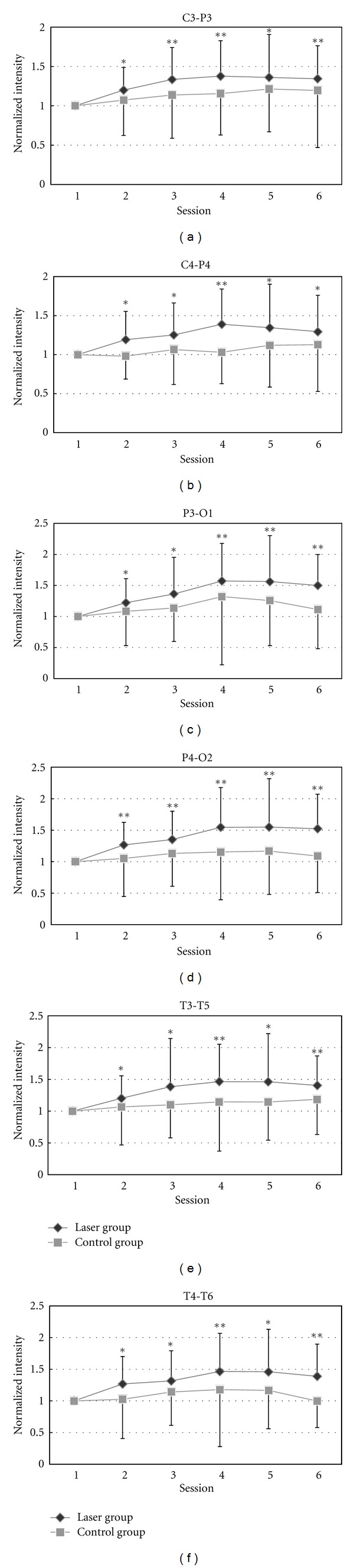
The statistic analysis of the alpha band (8–13 Hz) by comparing the baseline and each session in laser and placebo group is shown in different areas: (a) C3-P3, (b) C4-P4, (c) P3-O1, (d) P4-O2, (e) T3-T5, and (f) T4-T6. Paired-sample *t*-test on the alpha band power of EEG, comparing each session with the first session (baseline). *P < 0.05 by paired-sample *t*-test. **P < 0.01 by paired-sample *t*-test.

The average power of each session was normalized by dividing the corresponding data in the first session. The comparisons of alpha band power at six recording sites (C4-P4, P4-O2, T4-T6, C3-P3, P3-O1, and T3-T5) between the baseline and each session in the laser and the control group are shown in Figures 3(a), 3(b), 3(c), 3(d), 3(e), and 3(f). As compared with the baseline data (session 1), the alpha band power in the laser group increased significantly in the following sessions (2 to 6) at all the recording sites. Although there are some variations in the alpha band power in the control group, no statistically significant difference was found. 

**Figure 4:**
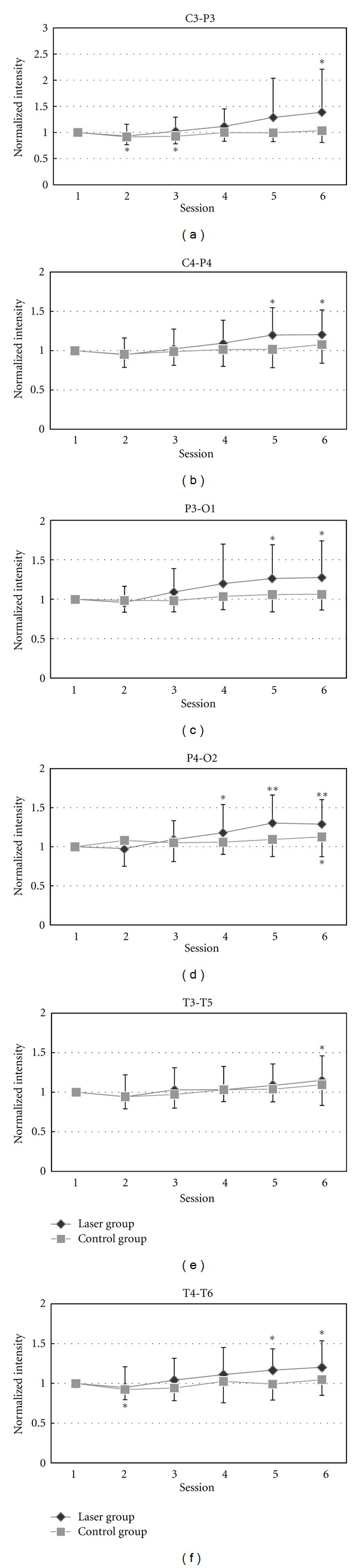
The statistic analysis of the theta band (4–7 Hz) by comparing the baseline and each session in laser and placebo group is shown in different areas: (a) C3-P3, (b) C4-P4, (c) P3-O1, (d) P4-O2, (e) T3-T5, and (f) T4-T6. Paired-sample *t*-test on the theta band power of EEG, comparing each session with the first session (baseline). *P < 0.05 by paired-sample *t*-test. **P < 0.01 by paired-sample *t*-test.

**Figure 5:**
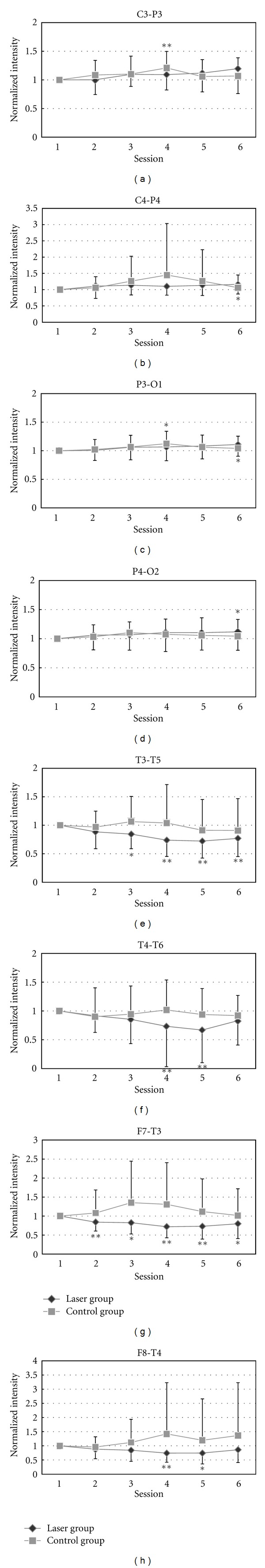
The statistic analysis of the beta band (13–50 Hz) by comparing the baseline and each session in laser and placebo group is shown in different areas: (a) C3-P3, (b) C4-P4, (c) P3-O1, (d) P4-O2, (e) T3-T5, and (f) T4-T6. Paired-sample *t*-test on the beta band power of EEG, comparing each session with the first session (baseline). *P < 0.05 by paired-sample *t*-test. **P < 0.01 by paired-sample *t*-test.

The statistic analysis of the delta band power showed no significant difference between corresponding data before and after in either laser or control groups. The band power of theta activities increased significantly (*P* < 0.05) during some of sessions 4–6 in the laser group (Figure 4). However, the band power of beta (13–50 Hz) activities from F8-T4, T4-T6, F7-T3, and T3-T5 after laser stimulation decreased significantly in the laser group (*P* < 0.05) as shown in Figure 5. 

## 4. Discussion

Acupuncture is an important treatment modality in traditional Chinese medicine. Acupuncture is effective for pain relief [17], stiffness, and disability in knee osteoarthritis [18, 19], and also chronic neck pain [20]. The modern acupuncture techniques include needle acupuncture, acupressure, electroacupuncture, and laser acupuncture [21]. The above-mentioned techniques are all applied to stimulate the so-called acupoint. For people afraid of painful needling or unable to tolerate the tingling of classical needle puncture, laser acupuncture is an ideal alternative. Laser acupuncture, a kind of phototherapy, has similar clinical effect to classical needling [22]. However, some important parameters in laser acupuncture, such as the wavelength, operation frequency, and dosage, are still not well delineated. 

 LLL stimulation can induce some biological or physiological changes. *In vitro*, irradiation with He-Ne laser could increase ATP level in cells [23], and the absorbance of living cells changed after laser irradiation [24]. In rats, increased endorphins were observed after laser irradiation at the Hoku acupoint [25]. *In vivo*, laser acupuncture at 10 Hz to the Neiguan acupoint could increase vagal activity and suppress cardiac sympathetic function [26]. In laser therapy, physiological changes have been described including increased urinary level of degradation product of serotonin [11], decreased inflammatory response [8, 27], and changes in blood circulation [28]. 

 The biological effects of LLL stimulation on the cell organism differed from those of high-intensity laser [29]. Although of lower energy (e.g., 20 Joules/cm^2^ in the present study), LLL was still able to deliver significant energy to induce some physiological effects. The near infrared wavelength laser ranged from 700 to 1400 nm can infiltrate the skin more than 2–5 mm, and its effect can be accumulated if irradiating at the same site [30]. Near infrared laser can induce more physical vibration or rotational motion than chemical reaction [30]. In the present study, we used a near infrared laser at a wavelength of 830 nm.

Laser-induced functional changes in the brain have rarely been described. Siedentopf et al. reported significant changes in brain activity in the ipsilateral hemisphere by radiating the Xiaxi (GB43) acupoint with a continuous laser beam [31]. With infrared laser stimulation at 10 Hz at the Yongquan (K1) acupoint of the left foot, the fMRI revealed active changes in the left primary motor cortex, left middle temporal gyrus, and the bilateral cuneus gyri [15]. Using modulation wave and continuous wave laser to irradiate the Yongquan acupoint, different stimulation frequencies could lead to activation at different regions of the brain [15]. These findings indicated that changes in brain activation caused by laser stimulation occurred in either cerebral hemisphere and seemed not only to have an endogenous effect of primary sensory conduction. 

Brain activation from laser stimulation outside the specific acupoint, such as stimulation at the palm in the present study, had so far not been reported. In the present study, the main EEG changes induced by insensible LLL stimulation at the palm outside the acupoint included amplitude activation of the alpha rhythms during and after stimulation; amplitude activation of theta waves after stimulation; amplitude reduction of beta activities during and after LLL stimulation. In view of the normal distribution of various frequency bands of EEG activities, it is reasonable that the laser-induced amplitude changes in alpha rhythms and in beta activities occur in the posterior and the anterior part of the head, respectively. However, we also found unusual prolonged EEG amplitude changes 15 minutes or possibly longer after termination of laser stimulation. 

 In normal, awake, relaxed adults with eye closure, the dominant brain waves are alpha rhythms distributed mainly in the posterior head regions. The alpha rhythms are attenuated by visual attention, mental efforts, and also drowsiness [32]. To obtain better presentation of alpha rhythms, it seems better to let the subjects keep their eyes closed. However, in designing the procedures for the present study, we performed and completed the whole 30-minute recording period in several volunteers with eye closure and found them difficult to keep fully awake but easy to fall into drowsiness. To prevent drowsiness, all the subjects in either the laser or the control group were therefore required to keep eye opening throughout the whole test period. As the appearance of theta waves is considered one of the important hallmarks of the onset of drowsiness [33] and no significant activation of theta waves was found in the control group (Figure 4), we undoubtedly believe that our study design was appropriate to keep the tester fully awake.

When stimulating at the skin, high-intensity laser may cause local heat or pain [34]. Perception of heat or pain is then able to evoke not only time-locked cerebral event-related potentials but also transient changes of the ongoing EEG activities. However, Iannetti et al. proposed that indirect readout of central nociceptive processing plays an important role in laser-evoked brain response [35]. In the present study, we found that EEG activation following stimulation with insensible LLL, that is, the tester in the laser group during EEG recording, did not feel heat or pain at all. The specific neural structures and pathway involving in the LLL-induced EEG changes need further clarification.

In classical acupuncture, needling correctly at the acupoint is essential to obtain a satisfactory therapeutic effect. Acupuncture stimulates the somatic afferent nerves of the skin and underlying muscles at the acupoint [36]. The sensory information is carried to the sensory cortex through the specific somatosensory pathway, as well as to other cortical areas through the nonspecific ascending neural pathways. Neurochemical alterations following laser-induced neural processes may also play an important therapeutic role [36]. In treating pain of cervical spondylosis, Dong and Lin reported a definite or even better therapeutic effect with acupuncture outside the classical acupoint [37]. Thus, amplitude changes of brain waves induced by LLL stimulation at the palm (not at the acupoint) do not seem impossible.

In a comprehensive review of EEG studies of meditation, Cahn and Polich summarized that an overall slowing occurred subsequent to meditation with theta and alpha activation (meditation state). There were also some long-lasting EEG changes (meditation trait) in the meditator irrespective of being actively engaged in meditation [38]. They also concluded that some positive psychological and clinical effects could be obtained by meditation practice. As both the concurrent (state) and prolonged (trait) EEG activations were also observed in our subjects with LLL stimulation, we postulate that some psychological and clinical effects may result from long-term practice with LLL stimulation. However, further delicate and confirmatory study is necessary.

## 5. Conclusion

The effects of LLL stimulation at the palm on the different frequency bands of electroencephalogram (EEG) were investigated. Laser stimulation at 10 Hz can increase the power of alpha rhythms and theta activities in the occipital, parietal, and temporal regions. The effect can last at least 15 minutes after cessation of laser irradiation. An increase of alpha band and decrease of beta band following laser stimulation can be comparable to those EEG effects of practicing medication. In the future, LLL radiation can possibly be applied to clinical therapy, for example, insomnia.

## Figures and Tables

**Table 1:** The comparison of average power in the 1st session for alpha band (8–13 Hz).

	Laser group *n* = 20	Control group *n* = 20	*F* value	*P* value
Fp2-F4	0.390 ± 0.343	0.434 ± 0.320	0.312	0.674
F4-C4	0.200 ± 0.152	0.191 ± 0.128	0.2262	0.841
C4-P4	0.169 ± 0.195	0.189 ± 0.173	0.000	0.733
P4-O2	0.517 ± 0.704	0.411 ± 0.340	1.799	0.546
Fp1-F3	0.330 ± 0.268	0.395 ± 0.282	0.609	0.459
F3-C3	0.203 ± 0.169	0.155 ± 0.110	3.857	0.300
C3-P3	0.197 ± 0.228	0.181 ± 0.182	0.274	0.808
P3-O1	0.358 ± 0.362	0.317 ± 0.236	0.963	0.763
Fp2-F8	0.412 ± 0.437	0.402 ± 0.280	0.423	0.935
F8-T4	0.198 ± 0.165	0.201 ± 0.105	0.875	0.946
T4-T6	0.293 ± 0.428	0.311 ± 0.224	0.075	0.869
Fp1-F7	0.404 ± 0.569	0.394 ± 0.292	0.157	0.942
F7-T3	0.264 ± 0.333	0.185 ± 0.086	2.209	0.310
T3-T5	0.219 ± 0.158	0.317 ± 0.356	3.465	0.268

Data are expressed as means ± standard deviation (*μ*v^2^).

**Table 2:** The comparison of average power in the 2nd session for alpha band (8–13 Hz).

	Laser group *n* = 20	Control group *n* = 20	*F* value	*P* value
Fp2-F4	0.403 ± 0.353	0.377 ± 0.315	0.008	0.807
F4-C4	0.199 ± 0.169	0.190 ± 0.151	0.000	0.806
C4-P4	0.171 ± 0.177	0.172 ± 0.139	0.139	0.976
P4-O2	0.535 ± 0.548	0.390 ± 0.350	5.072	0.326
Fp1-F3	0.319 ± 0.290	0.374 ± 0.335	0.672	0.578
F3-C3	0.176 ± 0.154	0.162 ± 0.136	0.390	0.771
C3-P3	0.187 ± 0.182	0.182 ± 0.178	0.103	0.937
P3-O1	0.372 ± 0.304	0.308 ± 0.231	2.867	0.457
Fp2-F8	0.378 ± 0.415	0.361 ± 0.290	0.195	0.885
F8-T4	0.171 ± 0.109	0.193 ± 0.118	0.478	0.544
T4-T6	0.276 ± 0.239	0.311 ± 0.333	0.502	0.709
Fp1-F7	0.394 ± 0.591	0.362 ± 0.331	0.298	0.831
F7-T3	0.234 ± 0.295	0.179 ± 0.104	1.147	0.441
T3-T5	0.253 ± 0.249	0.328 ± 0.412	2.647	0.487

Data are expressed as means ± standard deviation (*μ*v^2^).
